# Gut Microbiome and Osteoporosis

**DOI:** 10.14336/AD.2019.0523

**Published:** 2020-03-09

**Authors:** Kai Ding, Fei Hua, Wenge Ding

**Affiliations:** ^1^Department of Trauma Orthopedics, The Third Affiliated Hospital of Soochow University, Changzhou, China.; ^2^Department of Endocrinology, The Third Affiliated Hospital of Soochow University, Changzhou, China.; ^3^Department of Trauma Orthopedics, The Third Affiliated Hospital of Soochow University, Changzhou, China.

**Keywords:** Gut, Microbiome, osteoporosis, immunoregulation, bone, intervention

## Abstract

Gut microbiome refers to the microbes that live in human digestive tract and are symbiotic with the human body. They participate in the regulation of various physiological and pathological processes of the human body and are associated with various diseases. The pathological process of osteoporosis is affected by gut microbes. The molecular mechanisms of osteoporosis mainly include: 1) Intestinal barrier and nutrient absorption (involving SCFAs). 2) Immunoregulation (Th-17 and T-reg cells balance). 3) Regulation of intestinal-brain axis (involving 5-HT). Gut microbes can increase bone mass and improve osteoporosis by inhibiting osteoclast proliferation and differentiation, inducing apoptosis, reducing bone resorption, or promoting osteoblast proliferation and maturation. However, the therapeutic effect of gut microbes on osteoporosis remains to be further proven. At present, some of the findings on the impact of gut microbes on osteoporosis has been applied in clinical, including early diagnosis and intervention of osteoporosis and adjuvant therapy. In this article, we reviewed the molecular mechanisms underlying the regulatory effect of gut microbes on osteoporosis and the clinical practice of using gut microbes to improve bone health.

Gut Microbiome refers to microorganisms that are symbiotic in the human intestine [1]. They are originally obtained at birth, almost exclusively from the mother, and their composition can be affected by environmental factors such as age, diet, disease, travel and drug use. In adulthood, the composition of gut microbiome is relatively stable. The gut microbiome consists of about 1,200 species of bacteria, and the main taxa include Bacteroides, Firmicutes, actinomycetes, Proteobacteria and Verrucomicrobia [2]. The number of gut microbes is huge, which is more than the total number of cells in the human body [3]. Gut microbes not only participate in the regulation of various physiological functions of the human body, including physiological regulation of intestine, nutrient production and absorption, growth, energy balance, metabolic balance, immune function, brain behavioral function and inflammatory response, but also associate with some complex human diseases such as obesity, irritable bowel syndrome, type 1 and type 2 diabetes, colon cancer, Parkinson's disease, transient cerebral ischemia and rheumatoid arthritis [4-6].

Gut microbes grow in a nutrient-rich environment, and some bacteria are essential for maintaining the health of the host, such as improving energy extraction from food, eliminating pathogenic bacteria, and stimulating tissue production [7]. Gut bacteria have a beneficial effect on intestinal homeostasis by enhancing the proliferation and viability of intestinal epithelial cells and improving their barrier function [8]. In fact, mice raised under aseptic conditions exhibited many functional weaknesses and destroyed homeostasis of their bodies [9]. These observations indicate that there is an active, dynamic link between gut microbes and the host. More and more studies have shown that gut microbes and various human systems have a non-negligible correlation. For example, gut microbes may regulate inflammatory response and tumor-related diseases in the digestive system [10-15], they can delay the progression of neurological diseases [16], and they may prevent the occurrence and development of respiratory diseases [17-20].

## Gut microbiome is involved in the regulation of the bone homeostasis and the mechanism of osteoporosis

In the life of a person, bones are subjected to various stresses and strains, which may cause various bone injuries [21]. In order to maintain the integrity of the bone, the human body constantly remodels the bone, and in adults, 5-10% of the bone is renewed every year [21]. The bone renewal is a process involving co-coupled activation of a group of cells called bone remodeling units [22]. The bone remodeling cell unit contains four types of cells: osteoblast [22], osteoclasts [23], osteocytes [24]and endosteal cells [25]. The bone remodeling cycle consists of four distinct phases: initiation, resorption, reversal, and formation [26]. The dynamic balance of osteoblasts and osteoclasts runs through these four phases [26].

In women, the onset of menopause is a major risk factor of postmenopausal primary osteoporosis [27]. Decreased estrogen leads to two stages of bone loss: early rapid loss of trabecular and cortical bones due to increased number of osteocytes and reduced apoptosis of in these cells, and the second slower long-term bone loss due to reduced activity of osteocytes [27]. Secondary osteoporosis is caused by a variety of pathological factors, including smoking, type 1 diabetes (T1D), parathyroid disease, inflammatory bowel disease (IBD), arthritis and glucocorticoid therapy [28].

Bone is a dynamic organ that relies on the dynamic balance between osteoblasts and osteoclasts to maintain its normal function, and imbalance between osteoblasts and osteoclasts can lead to bone disease. Bone homeostasis can be regulated by estrogen, parathyroid hormone and immune cells [29]. Bone is also an important system of the human body, and the homeostasis of bone metabolism is closely related to intestinal flora [30]. Recent studies have shown that gut microbes can be a key regulatory factor in bone physiology[31]. In the following, we elaborated the role of gut microbes in the regulation of bone homeostasis and their impact on improving osteoporosis from physiological and pathological aspects, which involves intestinal barrier, immune system, and intestinal-brain axis.

## Intestinal epithelial mucosal barrier: nutrient absorption

Many intestinal floras can affect nutrient absorption. For example, in the intestinal tract, elevated concentrations of lactobacillus and bifidobacteria can promote the absorption of minerals such as calcium, magnesium and phosphorus, and thus increase bone mineral density (BMD) [32]. Studies have also shown that the composition of the gut microbiomes can affect the pH of the gut [33], which is very important for nutrient absorption, especially calcium absorption [34]. In addition, intestinal microbes play a vital role in the synthesis of vitamin B and K as well as the metabolism of bile acids [35]. Vitamin B and K are essential for bone health [36, 37], and bile acids may play a key role in the control of calcium absorption[38]. The gut microbiome helps break down macromolecules into smaller components that are easier to absorb, which is important for bone health and metabolism [39], and thus effectively alleviate or delay osteoporosis and increase bone density.

Nutrient absorption can be affected by host diet, which in turn affects the composition of the micro-organisms [40]. The uptake of carbohydrates and other nutrients provides energy for the survival of gut bacteria, and the composition of the diet has an important impact on the microbial community[41]. A high-calorie diet is associated with a reduced ratio of Bacteroides/Firmicutes [42], which may result in metabolic disorders in the host. On the other hand, low-calorie diet increases the concentration of harmful substances in the intestine [43], which may also have a negative impact on host health. Although adequate protein intake provides necessary material for bone growth, excess protein in the diet may cause elevated levels of toxins, such as hydrogen sulfide and methane, in the gut [44].

**Figure 1.**
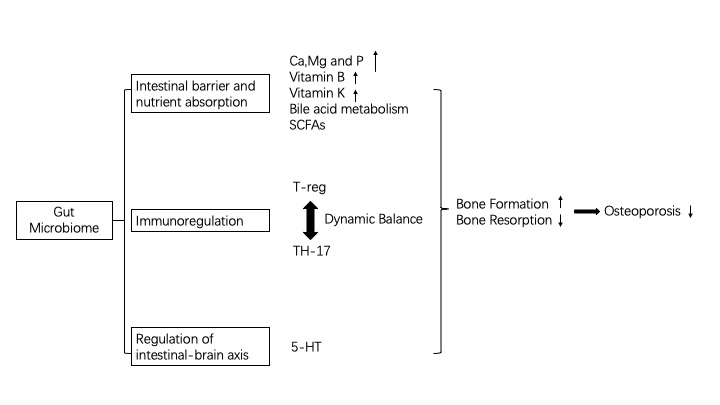
Molecular mechanism of Gut Microbiome regulating Osteoporosis.

Studies have found that microbial fermentation of dietary fibers produces short-chain fatty acids (SCFAs) that are regulators of osteocyte metabolism and bone mass. Feeding mice with SCFAs and a high-fiber diet can markedly increase bone mass, prevent bone loss, and significantly improve osteoporosis. The mechanism of the protective effect of SCFAs on bone mass lies in that SCFAs regulate the differentiation of osteoclast and inhibit bone resorption in vitro and in vivo without affecting bone formation. Specifically, both propionic acid (C3) and butyric acid (C4) are short-chain fatty acids. They can induce metabolic remodeling of osteoclasts, causing oxidative phosphorylation in glycolysis, which reduces the expression of osteoclast-associated genes, such as TRAF6 and NFATc1 [45], leading to the inhibition of osteoclast differentiation and the reduction of bone resorption. These data indicate that SCFA is a key regulator of osteoclast metabolism and bone homeostasis. Insulin-like growth factor 1 (IGF-1) is an important hormone that affects bone growth [46], and its serum level is significantly increased in microbial colonization reactions when the production of IGF-1 in the liver and adipose tissue was significantly increased. In contrast, serum IGF-1 is dramatically reduced in mice after antibiotic treatment, leading to the inhibition of bone formation [47]. SCFA supplementation in mice treated with antibiotics can restore serum IGF-1 and bone mass to the levels equivalent to those in mice that were not treated with antibiotics [47].It indicates that microbiota produced SCFAs may promote the production of serum IGF-1. Studies have shown that the gut microbiome may be an anabolic stimulus to the bone that act through IGF-1[47, 48]. Thus, manipulation of the microbiome or its metabolites may improve osteoporosis. Intestinal microbes regulate the homeostasis of the bone both positively and negatively. Recent studies have found that high fat diet (HFD) reduces the number of long-term LinSca-1^+^c-Kit^+^ (LSK) stem cells and shifts lymphoid cells to myeloid cell differentiation. HFD may impair the function of the bone marrow micro-ecological environment, leading to poor reorganization of hemato-poietic stem cells [49]. HFD induces PPARg2 activation, enhances bone marrow lipogenesis, and impairs osteoblast formation. These effects can be transferred from high-fat diet mice to healthy mice by gut microflora adoption through fecal transplantation [49], which can cause osteoporosis in normal healthy mice. Therefore, maintaining a balanced diet and proper ratio of dietary fiber, starch, and protein is critical for bone health because proper dietary uptake can cause positive changes in gut microbiome and promotes the absorption of nutrients by the intestinal epithelial mucosal barrier, which is beneficial for skeletal metabolism. On the contrary, imbalanced diet may negatively affect the bone metabolism, leading to osteoporosis. However, excessive SCFA uptake can have an adverse effect on the body. Zumbrun et al. found that in mice with high-fiber diet, the butyrate production in the intestine of mice was significantly increased, accompanied by a marked increase in the susceptibility of the mice to pathogenic Escherichia coli. O157:H7, and the mechanism maybe that the intestinal tissue of mice with HFD bound more Stx1(Shiga toxin 1) and expressed more globotriao-sylceramide [50] (Fig. 1).

## Immune System: Helper T-cell 17 (Th-17) and regulatory T cells (T-reg)

Recent data suggest that osteoporosis and inflammatory joint disease share a common immune component. In rheumatic diseases and estrogen deficiency, the activation of CD4^+^ T cells is enhanced, which increases the production of pro-inflammatory factors and osteocytic factors such as IL-17, TNF-α, IL-1β and RANKL [51-53]. Dysbiosis may change the immune response in the intestinal and alter the migration of monocytes and lymphocytes in tissues, including bone marrow [54]. The number of monocytes and osteoclasts in the bone marrow is reduced in sterile mice, but is restored to the normal level after colonization of gut microflora [54]. Adoptive transfer of gut microbiomes can cause changes in monocytes [55]. Crohn's disease is often associated with severe bone loss and dysbiosis. Th-17 cells can migrate to the bone marrow and recruit osteoclast precursors, resulting in massive osteoclastogenesis [56]. In normal mice, osteoclasts induce the production of T-reg cells, and newly produced osteoclasts can activate TNF-α-producing CD4+ T cells [57].

Gut microbiome can directly regulate the immune responses of the host, and the dynamic balance of Th-17 cells and T-reg cells has always been a hot topic in immunology research. The relationship between Th-17 cells and T-reg cells is very complex. It can be either inhibitory or promoting. The balance between Th-17 cells and T-reg cells is critical for inflammation reaction and the regulation of tumor metabolism [58]. Some studies indicate that the immune regulatory function of gut microbiome may be regulated by Th-17/T-reg cells. First, Littman and his colleagues demonstrated that the mouse Th-17 cells express a SFB-specific T cell receptor (TCR) in response to segmented filamentous bacteria (SFB) that are commensal bacteria in mice [59]. It is surprising that a gut flora that does not destroy intestinal epithelial cells can program immune cells that are specific to it. This phenomenon was also found in Helicobacter pylori (Hp): Hp can have induce the expression of Hp-specific T-reg cells[60]. Belkaid and his colleagues also confirmed this result in their study. They demonstrated that the response of CD8+ T cells to skin symbiotic Staphylococcus strains is not only specific, but also lasts for several months, indicating that the programming effect of the bacterium is effective and long lasting [61]. Donkor et al. stimulated normal peripheral blood mononuclear cells as well as umbilical cord and spleen derived mononuclear/ macrophage with several intestinal probiotics and found that the anti-inflammatory and pro-inflammatory factors that were secreted by these cells were markedly increased. Among these intestinal probiotics, Bifidobacteria can promote mononuclear macrophages to secrete large amount of TGF-β, thereby inducing the differentiation of Th-17/T-reg cells [62]. In addition, lacking T-reg cells may lead to a deadly CD4+ T cell-driven autoimmune disease [58]. Lactobacillus reuteri can alter the metabolomic characteristics that are disrupted by T-reg cell defects and restores the level of inosine, a purine metabolite, primarily by reducing Th1/Th2 cells and their associated cytokines. Inosine itself can prolong life span and inhibit multiple organ inflammation. The underlying mechanism can be that the in vitro inhibitory effect of inosine on Th1 and Th2 cell differentiation depends on the adenosine A 2A receptor, which is also required for the function of inosine and L. reuteri in vivo [63].

T-reg cells have immunosuppressive ability, and they induce and maintain immune tolerance of the body. The transcription factor Foxp3 controls the development and functions of T-reg cells. T-reg cells can be divided into three types according to their origins: thymic T-reg cells (tT-reg), peripheral blood T-reg cells (pT-reg) and in vitro induced T-reg cells (iT-reg) [64, 65]. In the intestine, T-reg cells maintain a rich microbial community and promote food digestion. Their main function is to control microbial factors and the proinflammatory response of food factors in the intestinal tract. The intestine contains both tT-reg and pT-reg cells, but the immune tolerance in the intestine is mainly mediated by pT-reg cells. Lack of pT-reg cells in the intestine causes an increase in harmful microbial communities and an increase in type 2 immunity. Gut microbiota dwell in the intestine and their abundance increases from the small intestine to the colon. Therefore, the population of pT-reg cells in the colon is greatly affected by the resident microflora. In a sterile mouse or a mouse treated with broad-spectrum antibiotics, the abundance of colonic pT-reg cells is significantly reduced [66-68], and transferring normal mouse gut microbial community to sterile mice can stably induce the production of pT-reg cells in the colon [68-70]. A wide range of bacterial genera can promote the production of pT-reg cells in the colon. These genera include Clostridium, Bacteroides, Bifidobacterium, Lactobacillus and Helicobacter [60, 69, 71-75]. Helicobacter species have been shown to drive the production of antigen-specific T-reg cells in the colon [60, 71]. Many studies have found that key bacterial drivers of antigen-specific T-reg cell responses are mucosa associated and thus their antigens are constantly exposed to the immune system [70]. In addition to providing antigens, bacteria also act as adjuvants to shape the T-reg cell response. Broad-spectrum antibiotics are much more potent in depleting RORγt+ T-reg cells than individual antibiotics [69]. Activation of the innate immune receptor TLR2 by bacterial components appears to be a common mechanism by which enteric bacteria promote T-reg cells, and Bacteroides fragilis A and cell surface β-glucan and galactan polysaccharides from Bifidobacterium bifidum have been shown to be present [76, 77]. Large poly-saccharides produced by H. hepaticus also signal through TLR2 and induce anti-inflammatory features in macrophages, including IL-10 production, which may affect intestinal Treg cell responses [78]. Notably, IL-10 production by T-reg cells appears to be particularly dependent on the gut microbiota, as IL-10 producing T-reg cells are considerably less in the colon of sterile or antibiotic treated mice. In addition, colonization of sterile mice with Clostridium strains or Bacteroides fragilis can increase the abundance of IL-10 + T-reg cells. Microbial populations also significantly enhanced the expression of the immunoregulatory receptor CTLA-4 in T-reg cells [74, 77]. In general, the microbiota plays an important role in promoting the maintenance and function of T-reg cells in the intestine, although there are many unknown mechanisms for how the microbiota regulates T-reg cell responses.

Short-chain fatty acids such as butyrate, propionate and acetate may also affect the intestinal T-reg cell response. Most SCFAs are produced through fermentation of dietary fiber by the gut microbiota. Due to the enrichment of Clostridium and Bacteroides in the large intestine, short-chain fatty acids are abundant in the large intestine and can induce T-reg cell responses [48]. There is substantial evidence that SCFAs promote pT-reg cell responses in the gut, particularly in the colon [79-81]. SCFA is thought to promote intestinal T-reg cells by two major mechanisms: recognition by certain G-protein-coupled receptors, such as GPR43 and GPR109A that are expressed by colonic T-reg cells, and colonic epithelial cells and innate immune cells, respectively, and the histone deacetylase-inhibitory activity of SCFAs [82].

Another dietary factor that affects intestinal T-reg cells is vitamin A. Vitamin A is a fat-soluble vitamin that is metabolized into its biologically active form of retinoic acid (RA) through a series of metabolic steps, including retinal dehydrogenase oxidation (RALDHs)[83]. Vitamin A is present in high concentrations in the intestine and is the core of pT-reg cell homeostasis in the gut [84]. In the presence of TGF-β1, RA induces the differentiation of pT-reg cells [85, 86]. In addition, CNS1, which is required for the generation of pT-reg cells, contains the binding site of retinoic acid receptor and isodimer of retinoid X receptor, which is activated by RA61. CD103 + DC is particularly well suited to promote T-reg cell responses in the gut through RALDH-mediated RA production and potential TGF-β1 activation by integrin αVβ8 [85, 87]. The development of RORγt+ pT-reg cells may depend on vitamin A. Mice fed a vitamin A-deficient diet or treated with RA receptor inhibitor show a decrease in the population of RORγt+ T-reg cells [70]. Therefore, vitamin A appears to specifically drive the production of RORγt+ T-reg cells in the gut. Other essential nutrients such as folic acid, niacin and branched-chain amino acids also can positively regulate T-reg cell responses in the gut [82, 84, 88].

T-reg cells are important regulatory cells in primary osteoporosis caused by estrogen deficiency. Estrogen can stimulate the proliferation and differentiation of T-reg cells, thereby inhibiting osteogenesis [58]. During this process, FOXP3 transgenic mice can avoid bone loss induced by ovariectomy [89]. The accumulation of T-reg cells at the highly remodeled osteophyte site can stimulate bone growth. The specific mechanism may involve the function of T-reg cells in osteoclast-mediated bone destruction: T-reg cells have immunosuppressive functions and can inhibit the differentiation of monocytes into osteoclasts [90]. Transplantation of CD4+CD25+ T cells into Rag knockout (T cell deficient) mice increases bone mass of the mice, which is associated with a decrease of the number of osteoclasts [91]. In addition, CD4+CD25+Foxp3+T-reg cells inhibit the formation of osteoclasts by producing IL-4 and IL-10 [89]. Human T-reg cells isolated from the blood can inhibit osteoclast differentiation by producing TGF-β and IL-4 [92]. After treatment with CD3 and CD28 antibodies, T-reg cells can express several cytokines that can inhibit osteoclast differentiation, such as GM-CSF, IL-5 and IL-10 [93]. TGF-β, IL-10, and IL-4 appear to be the major cytokines produced by T-reg cells that regulate osteoclastogenesis [94]. However, T-reg cells also can regulate osteoclast differentiation through cell-cell contact via cytotoxic T lymphocyte antigens [95, 96]. Recently, it has been reported that CTLA-4-induced osteoclastogenesis inhibition is through the CD80-86 pathway both in vivo and in vitro. Mechanically, CTLA-4 mediates a marked increase in the expression of IκB kinase (IKKa) and NF-κB-inducible kinase (NIK) in osteoclast precursors [95]. The non-canonical NF-κB pathway subsequently triggers the activation of indoleamine-pyrrole 2,3-dioxygenase (IDO), increasing the level of kynurenine, which is the major product of tryptophan catabolism, and eventually enhancing the apoptosis of osteoclasts through inducing osteoclast progenitors by IDO [91]. Therefore, we speculate that by regulating the dynamic balance of Th-17/T-reg cell, intestinal microbes can increase the secretion of anti-inflammatory factors such as TGF-β and IL-10, inhibit the proliferation and differentiation of osteoclasts, induce apoptosis of osteoclasts, and reduce bone resorption, thereby increasing bone mass and improving primary osteoporosis [51, 97, 98]. Similarly, intestinal flora may also regulate secondary osteoporosis. Zhang Jing *et al.* [99] found that Lactobacillus reuteri can prevent the inhibition of Wnt10b by activating anabolic pathways, thereby effectively inhibiting the bone loss in T1D mice. The specific mechanism is that in a high sugar environment, L. reuteri can inhibit the inflammatory response and reduce inflammation-caused inhibition of osteoblast activity, thereby reducing bone loss and alleviating osteoporosis caused by T1D. The latest treatment for inflammatory bowel disease (IBD) also involves intestinal probiotics, including Lactobacilli, Bifidobacteria, and Saccharomyces boulardii. These probiotics relieve IBD-caused osteoporosis and reduce bone loss through key regulatory factors of inflammation such as T-reg cells and SCFAs [100].

## Intestinal-brain axis: 5-hydroxytryptamine

In recent years, it has been found that the gut microbiota probably has an important influence on the nervous system through regulating hormones and neuro-transmitters such as 5-hydroxytryptamine (5-HT) [101]. The 5-HT signal transduction system is considered to play an important regulatory role in bone development and maintenance [102]. Bliziotes et al. reported that both osteoblasts and osteoclasts contain serotonin receptors, and increased level of serotonin is associated with decreased bone mass in mice [103]. Another study found that using synthetic molecular inhibitors to reduce 5-HT level can prevent bone loss caused by ovariectomy (OVX) [104]. There are two types of 5-HT: central and peripheral. 5-HT synthesized by brainstem spinal neurons acts in the central nervous system, mainly by activating five HT2C receptors on neurons in the ventromedial hypothalamus to promote bone growth, which increases the sympathetic nervous tone [104]. In mice, enhanced sympathetic nervous tone promotes the proliferation of osteoblasts and inhibits the proliferation and differentiation of osteoclasts in mice under the regulation of leptin [105]. The release of leptin from fat cells reduces the synthesis and excitability of 5-HT-producing neurons in the brainstem nucleus, thereby inhibiting the positive effect of central 5-HT on bone mass. The mechanism may involve the low-density lipoprotein receptor family (LRP5) gene and the transcription factor FOXO1. Up-regulation or down-regulation of LRP5 can cause dramatic changes in bone mass mainly through the negative regulation of tryptophan hydroxylase (TPH1) by LRP5, leading to increase or decrease of 5-HT in the peripheral circulation, thereby affecting bone mass [106]. FOXO1 is an important factor in intestine tract-mediated 5-HT action and osteoblast proliferation in mice [107]. Gut microbes have been shown to affect 5-HT synthesis by enterochromaffin cells (EC) and regulate the release of 5-HT [107]. Reigstad et al. found that SCFAs produced in the intestinal lumen, such as acetic acid and butyric acid, can increase the expression of Tph1 messenger RNA and the synthesis of 5-HT by EC [108]. When human intestinal microbes were transplanted into sterile mice, the 5-HT signal in these mice was changed: Tph1 mRNA expression and mucosal 5-HT content increased, whereas the number of EC and the expression of 5-HT vector was not affected, which indicates that the intestinal microbiota affects the function of EC through SCFA [108]. Therefore, the intestinal microbiome may regulate bone mass through 5-HT in the intestinal-brain axis, which provides a new idea for the treatment of osteoporosis. Clinical diagnosis and treatment of osteoporosis using gut microbiome

The impact of gut microbiome on osteoporosis has also been investigated in clinical studies. Researchers have analyzed the microflora in feces of female osteoporosis patients and the correlation between the gut microflora and estrogen levels in patients, and found that changes in gut microbial species are associated with estrogen level changes in patients which may create a new approach to prevent osteoporosis [107, 109]. However, a randomized, double-blind, placebo-controlled clinical trial initiated by Nilsson et al. found that providing L. reuteri (ATCCPTA 6475) to elderly people with reduced bone density resulted in bone mass increase, but the effect was not statistically significant [110]. Therefore, using L. reuteri to prevent bone loss in the elderly should be further verified. Lambert et al. found that oral probiotics combined with red clover extract (enriched with isoflavone aglycone) can significantly reduce bone loss caused by estrogen deficiency, improve osteoporosis, promote the production of beneficial estrogen metabolite and stimulate the production of estrogen. Noteworthy, supplementation of the probiotics + red clover extract complex together with calcium, magnesium, and calcitonin is more effective than supplementation of the complex alone [111]. Therefore, probiotics and prebiotic complexes in combination with bone mineral matrix can be a potential new treatment for osteoporosis.

## Application and prospect of using intestinal microbes to improve bone health

A growing number of studies have shown that gut microbiomes can combine with other factors, such as diet, genetic susceptibility, lifestyle and drugs, to improve the bone health under physiological and disease conditions. The gut microbes can activate inflammatory responses in various tissues including bone marrow by bacterial modifications or through the actions of their metabolites [112]. The mechanisms underlying the beneficial effect of gut microbiome are very complex and requires further study. At present, restoring the balance of the intestinal flora is considered a treatment method for various diseases. Specifically, the balance of intestinal flora can be restored by changing dietary habits and supplementing probiotics or their metabolites such as SCFAs, oligosaccharides, carbohydrates and dietary fiber. These substances can promote growth, alter the composition of intestinal microbes, stimulate anti-inflammatory responses, promote intestinal absorption of calcium, thereby increasing BMD. Oligosaccharides obtained from dairy products also have similar benefits. Various subtypes of Lactobacillus and Bifidobacteria have anti-inflammatory effects, and they can enhance vitamin D absorption and reduce osteoclast differentiation, thereby preventing bone loss caused by ovariectomy in mice [52, 53, 108]. Intestinal flora transplantation technique has been widely used in mice and gut microbiomes have been demonstrated to be involved in many diseases, including those that affect bone health. In humans, intestinal flora transplantation has been successfully used to treat intestinal diseases such as drug resistant bacteria-caused colitis. As a potential therapeutic approach, flora transplantation has gained more and more attention. Further studies are required to elucidate the relevant mechanisms of the beneficial impact of flora transplantation and to validate the therapeutic efficacy of this approach in bone diseases.
